# The Potential of a Stratified Approach to Drug Repurposing in Alzheimer’s Disease

**DOI:** 10.3390/biom14010011

**Published:** 2023-12-21

**Authors:** Chloe Anderson, Magda Bucholc, Paula L. McClean, Shu-Dong Zhang

**Affiliations:** 1 Personalised Medicine Centre, School of Medicine, Altnagelvin Hospital Campus, Ulster University, Glenshane Road, Derry/Londonderry BT47 6SB, UK; anderson-c25@ulster.ac.uk; 2 School of Computing, Engineering and Intelligent Systems, Magee Campus, Ulster University, Northland Road, Derry/Londonderry BT48 7JL, UK

**Keywords:** stratified medicine, personalised medicine, drug repurposing, Alzheimer’s disease

## Abstract

Alzheimer’s disease (AD) is a complex neurodegenerative condition that is characterized by the build-up of amyloid-beta plaques and neurofibrillary tangles. While multiple theories explaining the aetiology of the disease have been suggested, the underlying cause of the disease is still unknown. Despite this, several modifiable and non-modifiable factors that increase the risk of developing AD have been identified. To date, only eight AD drugs have ever gained regulatory approval, including six symptomatic and two disease-modifying drugs. However, not all are available in all countries and high costs associated with new disease-modifying biologics prevent large proportions of the patient population from accessing them. With the current patient population expected to triple by 2050, it is imperative that new, effective, and affordable drugs become available to patients. Traditional drug development strategies have a 99% failure rate in AD, which is far higher than in other disease areas. Even when a drug does reach the market, additional barriers such as high cost and lack of accessibility prevent patients from benefiting from them. In this review, we discuss how a stratified medicine drug repurposing approach may address some of the limitations and barriers that traditional strategies face in relation to drug development in AD. We believe that novel, stratified drug repurposing studies may expedite the discovery of alternative, effective, and more affordable treatment options for a rapidly expanding patient population in comparison with traditional drug development methods.

## 1. Introduction

In 1901, Auguste Deter, a 51-year-old female patient was admitted to a German asylum after becoming increasingly jealous of her husband and experiencing progressive memory loss. After her death in 1906, an autopsy was performed, and samples of her brain were sent to Alois Alzheimer who noted that there were “tangles of fibrils” in place of neurons and a “deposition of a special substance” throughout the cortex. Although Alzheimer noted that there was an increasing number of similar cases [1,2], the disease was not named until 1910 when Emil Kraepelin used the term “Alzheimer’s disease” in a medical textbook [3,4].

In over a century since this first case of Alzheimer’s disease (AD), we have identified that the “tangle of fibrils” and the “special substance” are the characteristic hallmarks of the disease: neurofibrillary tangles of hyperphosphorylated tau protein and amyloid-beta plaques. Despite our increasing understanding that AD is a complex neurodegenerative disease, the underlying aetiology remains unknown. Since 1901, the number of cases of dementia has risen to an estimated 55 million worldwide [5], with AD contributing to approximately 60–70% of dementia cases [5,6].

Despite this, treatment options for AD remain limited, with only four drugs on the market that target the symptoms of the disease being widely available [7]. Consequently, there remains an unmet need for effective, affordable, and widely available treatments that is not currently being met with traditional drug development strategies.

## 2. Pathophysiology of Alzheimer’s Disease

While the underlying aetiology of AD is unclear and theories that have been suggested remain largely unproven, pathophysiological understanding has increased in the last hundred years. At the macroscopic level, there is moderate cortical atrophy that often results in enlarged ventricles. The sulci of the frontal and temporal cortices appear to be widened, and the overall brain weight is decreased in patients with severe disease [8]. At the microscopic level, there is the formation of amyloid-beta plaques and neurofibrillary tangles [2], neuroinflammation [9], N-methyl-d-aspartate receptor overactivation [10], and a reduction in acetylcholine levels [11].

It has been suggested that amyloid-beta (Aβ) plaques are the earliest manifestation of the disease, which have been identified up to 15 years before disease onset [12]. Aβ plaques are formed from Aβ peptides that are derived from the proteolytic cleavage of amyloid precursor protein (APP), which is not itself neurotoxic. APP is processed via two pathways: the non-amyloidogenic pathway and the amyloidogenic pathway [13]. In the non-amyloidogenic pathway, APP is broken down by alpha- and gamma-secretase to produce a long-secreted form of APP (sAPPalpha) and C-terminal fragments (p3 and ACID50) that may enhance neuronal survival and improve memory formation [13,14]. In the amyloidogenic pathway, APP is cleaved by beta- and gamma-secretase to produce APP intracellular domain (ACID) and Aβ. Aβ has several isoforms that range from 39 to 43 amino acids in length. When the ratio of insoluble Aβ42 to Aβ40 rises, extracellular Aβ fibrils begin to form, which then aggregate to form plaques [15]. The amyloid hypothesis for the aetiology of AD was first proposed in 1991, suggesting that the build-up of Aβ is the underlying cause of disease [16]. However, some questions about this hypothesis have been raised as Aβ plaques are also found in some cognitively healthy individuals [17]. It is currently unclear if, given enough time, these individuals would go on to develop AD [18]. More research is necessary to establish whether the build-up of Aβ plaques is truly the underlying cause of the disease.

The neurofibrillary tangles found in AD are composed of tau protein, which in healthy individuals, plays a key role in the assembly and support of microtubules [19]. In AD, tau becomes abnormally hyperphosphorylated, allowing it to dissociate from the microtubule and aggregate into neurofibrillary tangles. Eventually, as more microtubules become destabilised, cell death occurs [20]. Some studies report that tau pathology is the first to appear in patients and that the development of tau tangles is what triggers the development of Aβ plaques [21]. However, conflicting findings report that Aβ plaques occur first, and this is what triggers the development of tau tangles [22]. A tau hypothesis explaining that tau is the causative agent of the disease has also been suggested [23].

A third theory suggests that the overactivation of N-methyl-D-aspartate (NMDA) receptors plays a key role in the development of AD [24]. Calcium homeostasis is necessary for regulating the energy production that maintains neuronal plasticity and synaptic transmission [25], and when functioning normally, NMDA receptors enable the correct levels of calcium to enter the cell, which allows for normal memory and learning [26]. However, in AD, there is an influx of Ca^2+^ through these receptors at rest, and this results in increased intracellular levels of calcium, which eventually leads to the loss of synaptic function and cell death due to excitotoxicity [24,27].

Neuroinflammation is also found in the brains of AD patients and appears to be caused by multiple factors. Normally, microglial cells monitor pathogens or degenerating neurons, but in AD, they become activated and are found at increased levels around plaques [28]. The initial acute inflammatory response is thought to help clear Aβ deposits, but factors such as pathological ageing and genetic mutations promote a sustained response from the microglia, leading to chronic neuroinflammation [9]. Chronically activated microglia produce proinflammatory mediators such as reactive oxygen species and cytokines, which further exacerbate inflammation, resulting in a vicious cycle of neurodegeneration and increased microglial activation [29,30]. As microglia become less able to clear Aβ deposits, peripheral macrophages may be recruited to help. However, it is likely that this further exacerbates neuroinflammation [31]. Over time, chronic inflammation results in structural changes in neurons, eventually leading to neuronal degeneration, and the inflammation both facilitates and exacerbates the development of plaques and tangles [9,31].

In healthy individuals, cholinergic neurons release acetylcholine into the synaptic cleft, which interacts with cholinergic receptors on post-synaptic neurons. Acetylcholine remains active until it is hydrolysed by either acetylcholinesterase or butyrlcholinesterase into choline and other by-products [32,33]. In AD, there is a severe degeneration of the cholinergic neurons in the Nucleus Basalis of Meynert, reducing the number of neurons from ~500,000 in the healthy brain to less than 100,000 in severe AD [34]. The remaining neurons exhibit a decreased level of choline acetyltransferase, leading to less acetylcholine being synthesised [33]. As acetylcholine is essential for learning, memory, and attention [35], the cholinergic hypothesis for the aetiology of AD suggests that the decrease in the level of acetylcholine is responsible for the development of the disease [11,34,36].

## 3. Factors Contributing to Alzheimer’s Disease

### 3.1. Non-Modifiable Risk Factors

Although none of the theories describing the aetiology of AD have been proven, several non-modifiable factors that increase the risk of developing the disease have been identified. Currently, the AD patient population is stratified into two groups based on age of onset. An arbitrary age of 65 years is typically used to determine strata, but as there is no definitive cut-off: this value may vary depending on the individual study [37]. Within these two groups, patients can be described as having either familial or sporadic AD; sporadic AD dominates and is associated with 90–95% of all cases [38,39,40,41].

For late-onset AD (LOAD), three major non-modifiable risk factors have been identified [42]. Age is the greatest risk factor for LOAD, with incidence and prevalence both drastically increasing with age. There are several ways in which ageing could contribute to increased susceptibility to AD, including through alterations in amyloid-beta metabolism [43,44], increased inflammation [45], and an increase in neurodegeneration coupled with a decrease in the ability to regenerate the white matter that has been damaged [46].

Sex is another non-modifiable risk factor, with women having a higher prevalence of AD than men (7.13% compared with 3.31%) as well as a higher incidence rate than men (13.25 compared with 7.02) [47]. Currently, it is unclear if women are also more susceptible to early-onset AD (EOAD) than men [48]. This higher incidence and prevalence may be partly explained by women on average living 4.5 years longer than men [49]. However, with 60% of all AD patients being post-menopausal women, it is possible that hormonal changes may also contribute [50,51,52]. In animal and in vitro studies, oestrogen was found to play several neuroprotective roles including promoting survival of cholinergic neurons, metabolism of APP through the non-amyloidogenic pathway, and via antioxidant properties [53]. Hormonal changes experienced in menopause can result in altered sleeping patterns [54] and cognitive dysfunction [55]. Individually, these symptoms are risk factors for AD, but in combination with decreasing oestrogen, they may explain why women are at a higher risk of developing the disease than men [50,52].

The apolipoprotein E (*APOE*) gene on chromosome 19 also plays a role in the development of LOAD [56]. There are three alleles of *APOE* in humans. *APOEɛ3* is the most common isoform and does not influence the risk of developing AD. *APOEɛ2* is the rarest isoform and is associated with a decreased risk of developing AD. *APOEɛ4* is associated with an increased risk of developing AD: one copy of this allele is associated with a three-fold increase, while two copies increase the risk of developing AD twelve-fold [57,58,59]. Neuroimaging studies appear to agree with this observation, having shown that *APOEɛ2* or *ɛ3* carriers have fewer plaques than *ɛ4* carriers [60].

Early-onset Alzheimer’s disease (EOAD) is considered to begin before the age of 65 years and contributes to an estimated 5–10% of all cases of AD [37]. The risk factors for EOAD are primarily genetic, with three genes responsible for 10–15% of familial cases [61]. Presenilin-1 and presenilin-2, encoded by *PSEN-1* and *PSEN-2*, respectively, are components of gamma-secretase [62]. While mutations in *PSEN-1* are more common, it has been suggested that mutations in either gene contribute to EOAD by altering the capabilities of γ-secretase, which results in increased production of Aβ42 [63,64]. Mutations in the amyloid precursor protein (*APP*) gene typically result in increased levels of plaque formation by increasing the amount of Aβ present, but the Icelandic mutation is protective and reduces levels of Aβ by up to 40% [65,66]. All three genes play a role in the amyloidogenic pathway, and mutations result in either more amyloid being produced or a higher ratio of Aβ42 being produced, leading to increased formation of Aβ plaques [67].

As only a small proportion of EOAD cases can be explained by mutations in *APP*, *PSEN-1*, and *PSEN-2*, and as a large proportion of remaining cases do not show a Mendelian inheritance pattern, it is highly likely that other genes are also involved including *SORL1* and *TREM2* [37]. Mutated forms of the sortilin-related receptor gene (*SORL1*) are thought to decrease the removal of Aβ peptides for clearance, while mutated forms of the triggering receptor expressed on myeloid cells 2 gene (*TREM2*) are thought to affect the amyloid and tau pathways [20,37,68,69].

### 3.2. Modifiable Risk Factors

In addition to the non-modifiable risk factors, several modifiable factors have also been identified. A meta-analysis by Livingston et al. [70] identified 12 factors that, if controlled, could potentially prevent or delay the onset of dementia in up to 40% of cases. This included actions such as controlling diabetes and hypertension, preventing head injuries, and reducing smoking, air pollution, and midlife obesity rates to reduce the build-up of neuropathological damage. Other actions such as treating hearing loss, maintaining an active social life, and achieving high levels of educational attainment were identified as helping to increase and/or maintain the cognitive reserve. Some actions including maintaining frequent exercise, reducing the occurrence of depression, and avoiding excess alcohol were identified as beneficial in both reducing neuropathological injuries and increasing and/or maintaining cognitive reserve. A combination of all these actions is needed to help reduce the number of people affected by dementia [70].

Obesity, specifically mid-life obesity, is a risk factor for developing dementia. There are several reasons for this including increasing insulin resistance and altered inflammatory pathways, which contribute to neuroinflammation [71]. Obesity also increases the risk of developing hypertension, which in and of itself is a risk factor for developing AD. It is thought that hypertension causes damage to the vascular walls, leading to hypoperfusion and ischemia, which increases neuroinflammation [72,73].

Obesity also increases the risk of developing type 2 diabetes, which is another risk factor associated with AD [74]. Type 2 diabetes is thought to contribute to AD in several ways; insulin resistance alters insulin signalling, which is normally neurotrophic and neuroprotective [75]. Hyperglycaemia may also contribute by altering neuronal activity and contributing to higher levels of Aβ in the brain. Diabetes also likely activates multiple inflammatory pathways and therefore contributes to and exacerbates the neuroinflammation occurring in AD [76].

Physical inactivity is also considered to be a risk factor for AD, with multiple studies identifying regular exercise as an easily modifiable way to reduce the likelihood of developing dementia or AD [77,78,79]. Studies have also found that those who regularly exercise have a larger hippocampus [80] and better spatial memory [81], while another study found that women who regularly walk are less likely to experience a cognitive decline [82].

One way to control some of these metabolic risk factors would be to encourage the uptake of the Mediterranean-DASH Diet Intervention for Neurodegenerative Delay (MIND). The MIND diet is a blend of the Mediterranean and the Dietary Approaches to Stop Hypertension (DASH) [83] diets, with a specific focus on increasing the consumption of green leafy vegetables and berries while decreasing the intake of animal products [84]. Multiple studies have shown that the MIND diet can slow the rate of cognitive decline and that a higher adherence to the MIND diet is associated with a lower risk of developing AD [84,85,86].

With treatment options for AD currently limited, public health initiatives to reduce the number of new cases of AD and dementia would be extremely beneficial. While a significant proportion of cases may be preventable, new treatments are still necessary for the remaining patient population.

## 4. Approved Pharmaceutical Interventions

Despite the vast and rapidly increasing patient population, the number of approved treatments for AD is limited, with only eight drugs ever gaining approval from a drug regulatory authority. Of these, only two are potentially disease-modifying, while the remaining six act only to alleviate some of the symptoms that patients experience [87,88,89,90,91,92,93,94]. Some drugs are not available worldwide and some have been removed from the market, further limiting treatment options for patients [95]. Approved drugs and associated mechanism of action, treatment type, disease stage, approval date, availability and cost are summarised in Table 1.

### 4.1. Symptomatic Therapies

#### 4.1.1. Cholinesterase Inhibitors

The oldest and largest class of drugs for AD are cholinesterase inhibitors, which work to inhibit the action of acetylcholinesterase and/or butrylcholinesterase to increase the concentration of acetylcholine in synapses [96,97].

Tacrine was first synthesised in 1945 with the aim of creating an antiseptic to treat wounded soldiers [98,99], but in 1980, it was discovered that it acted as a cholinesterase inhibitor [100]. Studies investigating its use in AD started in 1984, and it became the first approved drug for use in mild to moderate AD by the Food and Drug Administration (FDA) in 1993 [87]. Although studies suggest that tacrine provides small but significant benefits to patients, it does not alter the disease course [101,102]. Regardless, tacrine was withdrawn from the market in 2013 due to hepatotoxic side effects [103,104].

In 1983, Donepezil was developed in Japan [105], and in 1996, it was approved by the FDA for use in mild to moderate AD. In 2010, the FDA approved a higher dosage of 23 mg/day to treat moderate to severe disease [89]. Both tacrine and donepezil are reversible inhibitors, but, unlike tacrine, donepezil is highly specific and only inhibits the action of acetylcholine. Although the drug can provide mild relief from the symptoms of AD, cognitive decline continues, and the disease progression is not altered [106].

In 2000, the FDA approved rivastigmine, a pseudo-irreversible selective inhibitor of both acetylcholinesterase and butrylcholinesterase, to treat mild to moderate AD [107]. With gastrointestinal side effects being common with rivastigmine, administration via a transdermal patch is recommended [108].

Galantamine was first discovered in 1947 when it was isolated from the common snowdrop [109,110]. Synthetic forms were developed and first approved as a treatment for AD in Sweden in 2000 [88]. The FDA later approved it in 2001 [93]. Galantamine works in two ways: it acts as an inhibitor of acetylcholinesterase and as a potentiator of nicotinic and muscarinic acetylcholine receptors [103]. Similar to other cholinesterase inhibitors, galantamine only provides mild symptomatic relief and does not alter the course of the disease [95].

#### 4.1.2. N-Methyl-D-Aspartate Receptor Antagonist

Memantine is a non-competitive N-methyl-D-aspartate (NMDA) receptor antagonist that preferentially binds to open calcium channels. This reduces the influx of ions through the NMDA receptors and prevents the pathological build-up of Ca^2+^ and associated excitotoxicity [95]. Memantine is considered a second-line treatment and was first approved in Europe in 2002 and in the USA in 2003 [90,111]. Clinical studies have found that memantine provided small but significant benefits to patients [112,113]. However, similar to cholinesterase inhibitors, it does not modify disease progression [114].

#### 4.1.3. Sodium Oligomannate

In 2019, sodium oligomannate received conditional approval in China for use in patients with mild to moderate AD [115,116]. Sodium oligomannate is an oligosaccharide derived from brown algae that is thought to work by targeting multiple Aβ subregions and inhibiting the aggregation of Aβ [92]. It may also benefit patients by restoring the balance of the gut microbiome and reducing the levels of immune cells in the brain to help inhibit neuroinflammation. Reportedly, these effects protect synaptic integrity and have been found to improve cognition in vitro and in mouse models of disease [117]. Phase IV trials are currently being conducted in China [118], while a phase III trial recruiting patients across North America, Europe and China was suspended due to the impact of COVID-19 and will be restarted “at the right time” [119].

### 4.2. Disease Modifying Treatments

#### Monoclonal Antibodies

Aducanumab was first approved by the FDA in June 2021. It is a human immunoglobulin G1 monoclonal antibody that has been reported to cross the blood–brain barrier (BBB) and react with aggregated forms of Aβ to reduce the number of plaques in the brain [120]. The FDA’s decision to approve aducanumab has not been met without controversy due to its decision being largely based on two phase III clinical trials (EMERGE and ENGAGE) [91,121]. Biogen, the manufacturer, prematurely halted these trials, stating that they were unlikely to meet their endpoint goals [122]. However, several months later, Biogen stated that upon closer analysis of the data, the slowing of cognitive decline was clinically significant in the group receiving the highest drug dosage in one of the trials [123,124]. The FDA was advised by an independent panel of neurologists and biostatisticians that these results did not conclusively prove the benefits of the drug. Despite concerns, aducanumab received FDA approval through the accelerated approvals pathway in the United States of America [91,120,125,126,127].

The side effects of aducanumab are severe, with 35.2% of trial patients experiencing brain oedema and 19.1% experiencing microhaemorrhages [128]. As a result, additional costs and resources will need to be allocated to monitor for these effects in addition to the USD 28,600 price tag per patient per year for the drug [129,130]. With an estimated 60% of dementia patients living in low- and middle-income countries [131], this drug is unaffordable for many. Availability is also an issue. Due to the yet unproven results and severe side effects, the European Medicines Agency declined to approve the drug, stating that “the benefits of Adulhelm [aducanumab] did not outweigh its risks” [132,133]. The Japanese Health Ministry also declined approval in December 2021 due to inconclusive results [134]. In June 2022, Biogen withdrew its application for approval in Canada following an indication from Health Canada that its data were not sufficient to warrant approval [135].

Lecanemab is another G1 monoclonal human immunoglobulin antibody that targets soluble Aβ that has become aggregated. In a phase II clinical trial testing the drug in patients with early-stage AD, the 12-month endpoint goal of an 80% probability of at least a 25% reduction in the rate of cognitive decline was not met [136]. However, at 18 months of treatment, it was shown that there was a reduction in brain amyloid levels. The drug was generally well-tolerated, but like other amyloid-targeting drugs, some patients experienced micro- and macrohaemorrhages in the brain [136]. In May 2022, an application was made to the FDA based on phase II data. The FDA granted priority review status to the application and granted accelerated approval to the drug on 6 January 2023 [137,138]. Lecanemab then received traditional approval from the FDA on 6 July 2023 because a phase III trial showed that Lecanemab reduced amyloid burden in early-stage AD and was associated with a slower rate of cognitive decline [94,139]. Applications for approval have also been submitted to European, Japanese, and Chinese regulatory boards [140].

**Table 1:** Table summarising the approved pharmaceutical drugs available for Alzheimer’s disease patients. The cost of the cholinesterase inhibitors and memantine is variable due to the availability of generic versions.

Drug Name	Mechanism of Action	Treatment Type	Disease Stage	Common Side Effects	First Approval	Availability	Cost (per Year)	References
Tacrine	Cholinesterase Inhibitor	Symptomatic therapy	Mild to moderate	Hepatoxicity, liver enzyme elevations, gastrointestinal issues, dizziness, headache	1993	Withdrawn from the market in 2013	N/A (withdrawn from market)	[103,141]
Donepezil	Cholinesterase Inhibitor	Symptomatic therapy	Mild to moderate	Agitation, dizziness, gastrointestinal issues, hallucinations, headache, muscle cramps, sleep disorders, syncope	1996	90+ countries	~GBP £9.12–£1006.68	[95,103,142,143,144]
Rivastigmine	Cholinesterase Inhibitor	Symptomatic therapy	Mild, moderate, and severe	Anxiety, arrythmia, depression, gastrointestinal issues, headaches, hypertension, syncope, tremor	2000	80+ countries	~GBP £564.72	[95,103,143,145,146]
Galantamine	Cholinesterase Inhibitor	Symptomatic therapy	Mild to moderate	Arrythmia, asthenia, depression, dizziness, gastrointestinal issues, hypertension, hallucinations, headache, muscle spasms, syncope	2000	European Union, USA, Canada, Japan, and more	~GBP £478.80–£889.20	[95,103,143,147,148]
Memantine	N-methyl-D-aspartate receptor antagonist	Symptomatic therapy	Moderate to severe	Balance impairment, constipation, dyspnoea, headache, hypertension	2002	Europe, USA, Canada, China and more	~GBP £19.20–£698.16	[95,103,149,150]
Sodium Oligomannate	Amyloid-targeting, restoration of gut microbiome	Symptomatic therapy	Mild to moderate	Nasopharyngitis, haematuria, elevated liver enzymes and LDL cholesterol	2019	China	~USD $6200	[115,116,117,151]
Aducanumab	Amyloid-targeting	Disease-modifying	Mild	Amyloid-related imaging abnormalities	2021	USA	USD $28,600	[91,121]
Lecanemab	Amyloid-targeting	Disease-modifying	Mild	Amyloid-related imaging abnormalities	2023	USA	USD $26,500	[140,152]

## 5. Alternative Therapies

With so few treatment options available for patients, it is understandable that patients may turn to alternative therapies.

Huperzine A is an alkaloid that is isolated from the Huperzia Serrata plant. For centuries, Huperzia serrata has been used in traditional Chinese medicine to treat schizophrenia and memory loss with extensive studies identifying Huperzine A as the molecule that contributes to the effects of the treatment [153]. Huperzine A crosses the BBB and acts as a reversible and selective acetylcholinesterase inhibitor. There is also evidence that it acts as an NMDA receptor antagonist [154]. Several clinical trials have been conducted to determine the benefits of Huperzine A in the AD patient population, but the results have been mixed, with some finding that Huperzine A provides some benefit to patients [155,156,157], while another study found it was no better than placebo [158].

Currently, two medical foods have been specifically marketed for AD in the USA and/or Europe that aim to relieve symptoms of the disease. According to the FDA, medical foods are “a food which is formulated to be consumed…under the supervision of a physician and which is intended for the specific dietary management of a disease or condition for which distinctive nutritional requirements…are established by medical evaluation” [159].

The first medical food for AD was launched in 2009 under the brand name Axona, which aimed to target the metabolic deficiencies associated with AD. In early AD, brain glucose metabolism and utilization appear reduced [160], possibly due to a reduction in the expression of glucose transporters [161], forcing the brain to rely on other energy sources. Axona is a powder mix made from medium-chain triglycerides (MCTs), which can be metabolized into ketone bodies by the liver. It is thought that as ketone bodies can cross the BBB, they can act as an alternative energy source for the neuronal mitochondria [162,163]. Results from a clinical trial found some benefits for *APOEε4*-negative patients [164]. However, in 2013, Accera, the manufacturer of Axona, drew criticism from the FDA, who warned the company that their product was misbranded as a medical food as “there are no distinctive nutritional requirements or unique nutrient needs for individuals with mild to moderate Alzheimer’s disease” and instead, Axona should be classified as a drug [165]. In response, Accera set up a phase III clinical trial testing AC-1204, a product which has a similar mechanism to Axona, but a different formulation. Unfortunately, AC-1204 failed to improve cognition in patients with mild to moderate AD [166].

Souevenaid is another medical food created from a blend of docosahexaenoic acid, eicosapentaenoic acid, and choline combined with vitamins and minerals, which the manufacturer suggests “strengthens synapses to support memory function long term” [167,168]. A meta-analysis of clinical trials showed that Souvenaid led to improvements in verbal recall in patients with early-stage AD but had no beneficial effects in patients with later-stage AD [169]. However, other studies report that there are no benefits to patients [168,170].

In addition, other supplements and extracts claim to help with or prevent memory loss, but currently, the evidence to support such claims is lacking [171,172]. With the verdict on these alternative therapies being mixed, it is unlikely that they will address the need for targeted pharmaceutical interventions. However, under the supervision of a physician, some patients may benefit from alternative therapies alongside other treatment options.

## 6. Drugs under Development

As of January 2023, 141 drugs were being tested in 178 clinical trials, with the majority of these being disease-modifying [173].

Donanemab is an immunoglobulin G1 monoclonal antibody that targets an N-terminal truncated form of Aβ that is only found in established plaques. A phase III trial found that there is a significant slowing of clinical progression in patients and that after 1 year of treatment, 47% of patients receiving the drug had no disease progression in comparison with 29% receiving placebo. Similar to other amyloid-targeting drugs, amyloid-related imaging abnormalities were an associated side effect in patients [174]. An application for FDA approval has been submitted, and applications for approval with global regulatory boards are underway [175].

Gantenerumab is a fully human G1 immunoglobulin antibody that binds aggregated Aβ and clears plaques via Fc-mediated phagocytosis. A phase II trial found that subcutaneous doses of up to 1200 mg of gantenerumab administered every four weeks resulted in significant reductions in Aβ burden in prodromal to moderate AD patients. A follow-up from this study demonstrated that doses of the drug continued to reduce plaque burden up to 36 months after treatment started [176]. However, two phase III clinical trials, GRADUATE I and GRADUATE II, did not meet their clinical endpoint goals of slowing cognitive decline in patients with early AD [177].

Despite the significant number of drugs in the development pipeline, many will not progress to the clinic. Mirtazapine, an antidepressant, did not help with agitation in AD patients and was associated with a potentially higher mortality rate [178]. Solanezumab, a G1 monoclonal antibody that binds to Aβ, failed to significantly reduce cognitive decline in a phase III clinical trial [179]. Toriluzole, a new formulation of riluzole, which treats amyotrophic lateral sclerosis, did not perform better than a placebo in reducing brain volume loss or reducing the symptoms of cognitive impairment [180]. With so many failures in the search for a new treatment [181], it raises questions about why failure is seemingly the rule rather than the exception.

## 7. Why Do Drugs for Alzheimer’s Disease Keep Failing?

The failure rate for new drugs is extremely high in all disease areas, with an estimated 90% failing to ever make it to the market [182], but in AD, this is considerably higher, with over 99% of drugs failing to reach the market [181]. Between 2003 and 2019, no new drugs gained regulatory approval for use in AD anywhere worldwide, despite hundreds of clinical trials [183]. This has resulted in legitimate enquiry; why do drugs for AD keep failing and what can be done to overcome this?

As the exact cause of AD is yet to be identified, it is difficult to know exactly which pathological change should be targeted. Over the last decade, the most common target for new treatments has been Aβ plaques, but with different isoforms and multiple termini, choosing which to target to produce positive outcomes with minimal side effects has proven difficult [184]. It is also unclear how beneficial it is to exclusively target one aspect of the disease pathology. With increasing evidence that AD is a complex multifactorial condition [185], it is likely that a combination of therapies targeting multiple pathways or pathological changes will be needed.

It is also possible that drugs are being tested too late in the disease course. With evidence that pathological changes associated with AD begin 10 to 20 years before symptoms occur [8,12,186], it is possible that irreversible damage may have occurred in the brain by the time the patient is diagnosed. With many clinical trials only including symptomatic patients, it is possible that the disease is already too advanced for the drug, and this leads to findings appearing less significant than they are.

Misdiagnosis could play a role in the high rate of failure, particularly in patients with early-onset dementia (EOD) [187]. While AD is the most common cause of dementia, it only accounts for one-third of EOD cases. It is thought that many EOD patients are misdiagnosed, with anywhere from 30 to 50% receiving an inaccurate diagnosis [188]. An accurate, timely diagnosis is essential; cholinesterase inhibitors are beneficial in EOAD but worsen symptoms in frontotemporal dementia [189]. With diagnosis largely based on symptoms, family history, and memory assessments, it is understandable that the high degree of overlap between conditions leads to misdiagnosis [190]. However, for enrolment in clinical trials, a definitive method to determine the cause of a patient’s symptoms would ensure that the drugs are being tested on AD patients and that the results are not skewed by the inclusion of participants with other forms of dementia.

Although biomarkers are used in AD research settings, they are not routinely used in the clinical setting. Cerebral spinal fluid (CSF) biomarkers such as Aβ, phosphorylated tau, and total tau levels generally are not used due to the invasiveness of obtaining samples, while amyloid positron emission tomography (PET) imaging is not routinely used due to the cost [191]. While identifying various AD biomarkers that could be easily obtained would be exceptionally beneficial for diagnosing, stratifying, and staging patients, several potential issues need to be addressed first.

Firstly, the relationship between pathological burden and disease progression is unclear [192,193]. Evidence of Aβ deposits has been found up to twenty years before the onset of the disease [12], but the presence of Aβ plaques in the brain is not a guarantee of developing AD, as some cognitively healthy elderly individuals have been found to have high levels at autopsy [192]. Levels of tau pathology seem to correlate more closely with cognitive decline [194], but there are still exceptions to this [195]. With a lack of clarity on what level of plaque and tangles constitute AD, it makes it difficult to determine diagnostic and prognostic biomarker thresholds based solely on plaque and tangle pathology.

Even if we could identify what these thresholds might be, it is likely unwise to rely solely on biomarkers to diagnose patients, and instead, phenotypic data should be considered alongside this [193]. This is particularly relevant in elderly patients as pure AD pathology is seen in less than 30% of patients, with most patients having evidence of multiple proteinopathies [196]. It also raises questions about how to classify patients if they have positive biomarkers for AD and other neurological conditions. Should one disease be given a preferential diagnosis over the other [193]?

Investigation into why some patients have amyloid-beta and tau pathology or have copies of the *APOEɛ4* allele but are cognitively healthy could help identify protective and predictive biomarkers for AD. Predictive biomarkers would enable early pharmaceutical intervention before symptoms and irreversible damage occur and would also allow for targeted lifestyle interventions, such as introducing the MIND diet to help protect cognition [84]. Delaying or preventing AD is particularly important due to the lack of drugs that halt disease progression.

It would also be beneficial if we could identify prognostic biomarkers that could be used to determine which stage of the disease a patient is in. Currently, AD patients are placed into one of five categories: mild cognitive impairment (MCI), mild AD, moderate AD, moderately severe AD, and severe AD [197,198]. Determining these stages is largely based on cognitive assessments, such as the Mini-Mental State Examination [197], which can be influenced by factors such as prior educational attainment and, therefore, a patient may be easily placed into the wrong category [199]. Having a more definitive way to determine staging would be beneficial for clinical trials as it would allow manufacturers to determine if their drug is effective at different stages of the disease.

Currently, some clinical trials, particularly those looking at interventions in preclinical AD, use biomarkers such as Aβ-PET imaging and CSF biomarkers to determine whether a patient is eligible to take part. In addition to the cost and invasiveness of obtaining these biomarkers, the high rate of failure in identifying eligible participants is a pressing issue considering the already difficult nature of recruiting participants into trials. The identification of more easily obtained biomarkers (e.g., blood-based) could potentially encourage more participants to sign up for clinical trials, as it is likely that the invasive nature of CSF biomarkers discourages individuals from signing up [200,201]. This would potentially help to speed up the clinical trial process, a large proportion of which is spent on patient recruitment [202]. This has the potential to allow drugs to reach the market faster. In combination with better diagnostic biomarkers, enabling patients to be diagnosed earlier, and better prognostic biomarkers, which allow disease course to be more effectively monitored, could potentially benefit the lives of millions of patients globally.

## 8. Additional Barriers to Treatment

Even if a drug makes it to the market, there is no guarantee that it will be available to all patients. With most countries having their own drug regulatory boards, approval of a drug in one country does not guarantee approval in another. Aducanumab is currently available in the USA but has not been approved in Europe, Japan, or Canada [120,132,134,135]. While the full benefits of the drug have not yet been confirmed, its potential benefits are being limited to a small portion of the global AD patient population.

This is further complicated by the WHO’s estimate that 60% of dementia patients are living in low- and middle-income countries [5], resulting in the cost of drugs, particularly new biologics, being an issue. Aducanumab was initially marketed at USD 56,000 per patient per year [130], but in December 2021, Biogen, the manufacturer, reduced this to USD 28,600 per patient per year to improve access to the drug in the USA [129]. Despite this reduction in cost, the drug will still be unaffordable for many patients globally. Additionally, amyloid-targeting drugs commonly have microhaemorrhages and brain oedema as side effects, and patients may have to undergo additional tests to monitor these effects [120,128,130]. Not only will this add additional costs to an already expensive treatment, but it will need additional resources that may not be easily allocated or available, depending on the capability of the local healthcare system.

With the number of AD patients expected to rise [6], and limited treatment options on the market, it is essential that new treatment options are discovered. Additional barriers further reduce the number of drugs available to patients and with it likely being years before new drugs make it through the traditional drug development pipeline, there is a clear unmet need for new, effective, and affordable treatment options for the patient population.

## 9. Drug Repurposing

Some barriers and limitations could potentially be addressed by drug repurposing. Drug repurposing is a technique that finds new uses for drugs which have been approved for use in other conditions [203]. In comparison with traditional drug development, there are many advantages including a lower risk of failure, as the drug has already been proven safe for use in humans. Early clinical trial phases can potentially be bypassed due to pre-existing safety profiles, leading to a shorter development timeline and reduced costs [203,204].

Traditionally, drug repurposing has occurred accidentally, for example, thalidomide, an antiemetic drug, was approved to treat morning sickness in 1957 but was banned in most countries by 1962 due to its teratogenic effects [205,206]. In 1964, a patient presented with Erythema nodosum leprosum (ENL), a systemic condition that develops after using leprosy treatments for several years, and was prescribed thalidomide as a sedative. Within 48 h, all the patient’s skin lesions had cleared up. Following this, multiple clinical trials were conducted and found that thalidomide cleared up to 90% of skin lesions within days and completely resolved the condition within two weeks. Thalidomide was approved by the FDA to treat ENL in 1998 [203,207,208].

In silico pharmacology could be considered to have begun in the 1960s when the relationship between the pharmacokinetics, pharmacodynamics, and chemical structure of a drug was identified with computational means [209]. Since then, advances in computational power, allowing researchers to work with larger data sets and to run more resource-intensive algorithms with more efficiency and accuracy, have contributed to the development of computational approaches to drug repurposing. This has resulted in a more methodological approach to drug repurposing, with computational drug repurposing broadly categorized as either disease-based or drug-based [210,211].

Drug-based approaches rely on information about drugs, such as how a drug affects gene expression. The Connectivity Map (CMap) project was developed by The Broad Institute in 2006 and was the first Gene Expression Connectivity Mapping (GECM) software [212]. Since then, other GECMs such as sscMap [213], cudaMap [214], and QUADrATic [215] have been developed. GECM takes a gene signature containing a list of differentially expressed genes in a disease state and compares this against a library of expression profiles from small molecules. A matching algorithm then identifies which small molecules can enhance the effects of the gene signature as well as those that suppress the action of the gene signature and can therefore potentially be used to treat the disease [216]. This methodology has identified potential treatments for several disease areas including obesity [217], cancer [218], and osteoporosis [219].

Disease-based approaches rely on information about the disease. One approach involves phenotypic information. As the phenotypic expression of a drug’s side effects may share the same underlying pathways as the phenotypic expression of a disease, it is thought the phenome could be used to identify drug repurposing candidates. For this approach to work, an in-depth knowledge of molecular mechanisms is necessary [210,211].

However, drug repurposing is not without its limitations. In silico drug repurposing attempts are often limited by the library of drugs being tested. sscMap, for example, has a reference library of just over 1000 small molecules [213]. While it would be impractical, time-consuming, and expensive to test every available drug, by not doing so, it is likely that repurposing candidates are being missed. Once a drug-repurposing candidate has been identified and passed through the development pathways, existing patents can prevent it from being able to reach the market. It may also be difficult to patent a repurposed drug, and consequently, this may result in a lower profit margin for pharmaceutical companies, making drug repurposing less attractive than traditional development strategies [203].

Despite such limitations, drug repurposing has had success in other disease areas, particularly in cancer medicine [220]. Metformin, an antidiabetic drug, has been repurposed as an anti-cancer drug [221]. There are several pathways through which metformin works against cancer including through the activation of the PI3K-mTOR signalling pathway, which inhibits the proliferation of cancer cells with insulin receptor expression. It also activates the AMP-activated protein kinase pathway, which ultimately inhibits cell survival [222,223]. There have also been successes in other neurodegenerative conditions, such as Parkinson’s disease (PD). Amantadine, an anti-viral drug, was identified as a potential PD treatment after a patient with PD symptoms improved while on the drug and declined after stopping. This led to the first clinical trial of amantadine in PD in 1968 and FDA approval in 1973 [224,225,226]. There are three potential mechanisms identified through which this drug benefits PD patients: activation of pre- and post-synaptic dopamine systems, inhibition of NMDA receptors [227], and anticholinergic activity [228].

## 10. Drug Repurposing in Alzheimer’s Disease

**Figure 1:**
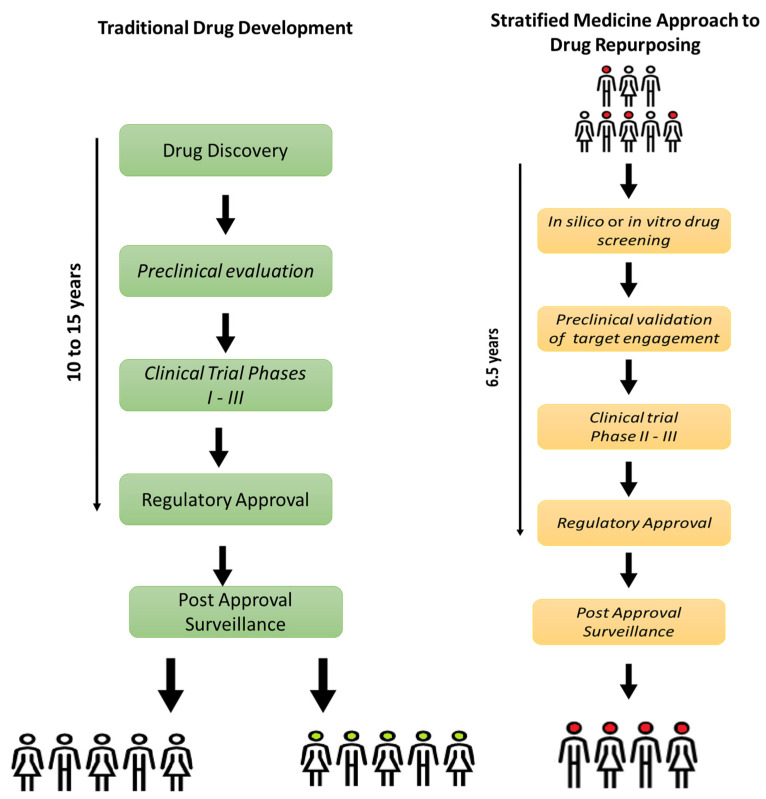
The traditional drug development pipeline (in green) takes an average of 10 to 15 years [183] with an associated average cost of USD 1.3 billion [230]. It starts with thousands of potential drug candidates and screens them out throughout the process. If a candidate makes it to regulatory approval, some patients will benefit from the drug, while others will not. In comparison, the stratified medicine drug repurposing pipeline will take considerably less time, with an average drug-repurposing candidate taking 6.5 years to make it to market and an associated cost of USD 300 million [231]. As the drugs are targeted towards specific strata of the patient population, patients are more likely to receive efficacious treatments with minimal side effects.

The benefits of drug repurposing could address the need for new more efficacious therapies in AD. As illustrated in Figure 1, drugs developed through the traditional pipeline take an average of 10–15 years [182] to create with an associated average cost of USD 1.3 billion [229]. As a result, drugs designed this way often have an exceptionally high price for manufacturers to recoup this initial investment. However, drugs developed via a repurposing pathway are on average approved after 6.5 years and have a much lower associated cost of USD 300 million [230]. Drugs developed in this way could be sold at a lower price, allowing a higher proportion of the patient population access. With 60% of dementia patients living in low- and middle-income countries [5], this is a moral imperative.

A shortened development timeline would also be beneficial. With an increasingly older population, it is estimated that by 2050, the number of AD patients will triple without any form of effective intervention [231]. A shortened development pipeline, coupled with pre-existing safety profiles, could result in drugs making it to market faster and therefore being available to patients earlier than drugs developed using traditional methods.

As drug repurposing candidates have already been approved for use in other conditions, their safety profile and side effects are known and well documented. This shortens the development pipeline and allows for a lower associated cost. Aducanumab was declined approval in multiple countries that stated that the side effects of the drug were too severe [132,134], but for repurposed drugs, a track record of safety and efficacy in humans is likely to increase the chance that the drug will be approved globally, allowing more patients to access potentially life-changing AD drugs.

Drug repurposing has already been attempted in AD with multiple methodologies used. Ballard et al. used a systematic literature review, combined with a Delphi consensus, to identify priority drug repurposing candidates including fasudil, a selective inhibitor of rho kinase 1 and 2 that acts as a vasodilator, phenserine, a cholinesterase inhibitor, and antiviral drugs [232]. A phase II clinical trial testing fasudil in AD is ongoing [233].

Grabowska et al. queried PubMed to identify drug repurposing articles related to AD published between May 2012 and May 2022. They included 124 relevant studies in their analysis, which identified 573 unique drug repurposing candidates. Clozapine, an anti-psychotic, was the most commonly identified candidate, appearing in six studies, while nine other drugs including adenosine, risperidone, tamoxifen, and verapamil were identified in five studies [234].

Kumar et al. utilised a virtual screening protocol that combined molecular docking, Prime/MM-GBSA calculations, and a BBB permeability filter with molecular dynamic simulations. Two candidates then underwent in vitro analysis due to a lack of pre-existing experimental evidence. They identified a total of six drugs with acetylcholinesterase inhibitory activity that could potentially be repurposed for use in AD [235].

Lee et al. developed a proteotranscriptomic-based computational drug repurposing methodology based on inverse associations between disease and drug-induced protein and gene perturbation patterns for use in AD. They identified bupivacaine, topiramate, selegiline, and iproniazid as potential drug-repurposing candidates [236].

Several clinical trials are currently evaluating drug-repurposing candidates for AD. Metformin, an antidiabetic drug, was identified as having neuroprotective effects [237]. However, results from clinical trials are mixed, with some studies showing that metformin reduces cognitive decline [238,239], while others have found that it could worsen cognitive health [240,241,242].

Lithium, a mood stabiliser, commonly used in psychiatric disorders such as bipolar disorder, has also been identified as a drug repurposing candidate for AD [237]. Lithium directly inhibits glycogen synthase kinase 3β (GSK3β) [243], and this is thought to be beneficial in AD as GSK3β plays a role in the hyperphosphorylation of tau [244]. Clinical trials have found that lithium provides benefits to AD patients through a decrease in cognitive decline [245]. More clinical trials are currently ongoing [246,247].

Sodium benzoate, which is a metabolite of cinnamon, a preservative, and is licensed by the FDA to treat urea cycle disorders, is another potential drug repurposing candidate for AD [238,248,249]. Some studies have been conducted on the benefits of sodium benzoate in AD and found that the drug was beneficial in small subsections of the patient population [250]. Notably, two studies found the drug to be most beneficial in female patients [249,251]. While more research is needed, evidence of differential efficacy in different populations shows that a more personalised approach to therapeutics may be needed in AD.

## 11. Stratified Medicine in Alzheimer’s Disease

Stratified medicine aims to offer the right treatment to the right patient at the right time. With such a heterogeneous patient population, it is possible that trying to target the population as a whole is resulting in many failures in drug development. Instead, stratifying the patient population and then targeting the more homogenous strata could prove beneficial.

To date, limited stratification has occurred in AD. For entry into some clinical trials, evidence of amyloid and tau biomarkers is required [252], but trials rarely go beyond this. A phase II clinical trial investigating the long-term safety and efficacy of Allopregnanolone, a neurosteroid metabolite of progesterone, is only recruiting *APOE ε4*-positive AD patients. While the study will not be completed until late 2026 [253], it will be interesting to see if this stratification leads to more positive outcomes.

Already, there is evidence of differential efficacy of the existing disease-modifying AD drugs based on the *APOE* genotype. A modest increase in efficacy has been noted in *APOE ε4* carriers, compared with non-carriers, when treated with amyloid-targeting drugs [254]. It is likely that the efficacy of future disease-modifying treatments may differ in sub-groups of the patient population and, as such, appropriate stratification could help identify patients who would gain the most benefit from emerging disease-modifying therapies.

There are several possibilities for stratification in AD. One such way would be to stratify the patient population into LOAD and EOAD. While considered to be the same disease, EOAD patients typically present with similar memory-related symptoms to LOAD but may experience a more aggressive disease progression and shorter relative survival time [12]. This is likely due to the presence of higher levels of plaque and tangles in comparison with LOAD patients [255]. Consequently, it is possible that EOAD patients require a more aggressive form of treatment to slow the rate of cognitive decline than LOAD patients.

EOAD patients could be further stratified based on genetic mutations. While *APP*, *PSEN-1*, and *PSEN-2* all play a role in the amyloidogenic pathway, they only account for 10–15% of familial cases of the disease [61]. With evidence that a substantial proportion of the remaining cases do not show a Mendelian pattern of inheritance [37], the identification of the genes involved would create new targets for drug development and drug repurposing, as well as a way with which to stratify the patient population.

The three major contributors to the development of LOAD could also be used to stratify the patient population. With postmenopausal women making up an estimated 60% of AD patients [256], stratifying the patient population by sex could prove beneficial [254]. Although more research is required, several genes have been identified as potentially having a sex-specific effect in AD. Female mouse models of AD, with mutations in *TREM2*, had increased microglial responses to stimuli and performed worse on memory-related tasks, while male mice with the same mutations had no such effects [257]. The brain-derived neurotrophic factor (*BDNF*) gene produces BDNF, a neuroprotective and neurotrophic factor that has a protective effect against Aβ. A SNP mutation in *BDNF* results in decreased BDNF secretion, memory loss, and a decrease in hippocampal volume. An association between this mutation and AD has been found in females but not in males [258]. Mutations in the *GRN* gene, which encodes for granulin protein that plays key roles in neuronal survival, are associated with AD in male patients but not in females [259]. It is possible that by stratifying the patient population by sex and targeting sex-specific genetic mutations, new treatments for AD could be identified for male and female patients.

*APOE* status is another way to stratify LOAD patients. With the different isoforms associated with differential risk for developing AD [58], it is possible that patients may benefit from different drugs, dependent on *APOE* status. Further stratification could also occur, as *APOE* appears to have differential effects based on sex and age. Women with *APOE ɛ3/ɛ4* have a higher risk of developing MCI and AD compared with men with the same genotype; however, men who are homozygous for *APOE ɛ4* are at a higher risk of AD than women who are homozygous for *ɛ4* [59,260,261]. *APOE* alleles also influence when a patient is likely to receive an AD diagnosis, with those who are homozygous for *ɛ4* being diagnosed at an average age of 68, those who are heterozygous for *ɛ4* diagnosed at an average age of 76, and non-carriers diagnosed at an average age of 84 [59].

Another option for the stratification of LOAD patients is by age, as the risk of developing AD increases with age. It is thought that as people age, the ability to clear Aβ decreases and neurodegeneration begins to happen faster than regeneration [59]. It is possible that there are some epigenetic changes that occur as a patient ages that also influence this risk [262] and therefore could function as a target for future interventions.

While more research is required to determine the optimal strategy to stratify the patient population and to identify future therapeutic targets, combining stratified medicine and drug repurposing strategies may be beneficial in AD. Although multiple drug repurposing studies have been conducted in AD, they tend to target the patient population as a whole, and this results in some of the same limitations that traditional non-stratified drug development faces. As such, an approach which combines stratified medicine and drug repurposing could address many of the barriers and limitations that traditional drug development and drug repurposing alone face. While drug repurposing addresses cost, availability, and time-to-market-related barriers, stratified medicine approaches will ensure more appropriate targeting of interventions. A stratified medicine approach to drug repurposing could reduce the failure rate of drug development in AD by targeting drugs to susceptible populations and thus preventing beneficial drugs from being misclassified as failures.

## 12. Conclusions

By combining stratified medicine and drug repurposing, some of the current challenges in drug development in AD could be addressed. While much work will be needed to fully address this, a stratified medicine approach to drug repurposing in Alzheimer’s disease has the ability to provide more effective and more affordable treatment options to a rapidly increasing patient population more quickly than traditional drug-development methods.

## Data Availability

Not Applicable.
